# Variability in clinical diagnoses during the ICD-8 and ICD-10 era

**DOI:** 10.1007/s00127-016-1265-9

**Published:** 2016-07-14

**Authors:** Julie Nordgaard, Kasper Jessen, Ditte Sæbye, Josef Parnas

**Affiliations:** 1 Early Psychosis Intervention Center, Region Zealand, University of Copenhagen, Smedegade 16, 4000 Roskilde, Denmark; 2 Center for Clinical Intervention and Neuropsychiatric Schizophrenia Research and Center for Neuropsychiatric Schizophrenia Research, Mental Health Center Glostrup, University of Copenhagen, Copenhagen, Denmark; 3 Institute of Preventive Medicine, Bispebjerg and Frederiksberg Hospital, University of Copenhagen, The Capital Region, Copenhagen, Denmark; 4 Center for Subjectivity Research, University of Copenhagen, Copenhagen, Denmark; 5 Mental Health Centre Hvidovre, University of Copenhagen, Copenhagen, Denmark

**Keywords:** Diagnosis, ICD-8, ICD-10, Uniformity, Clinical

## Abstract

**Aims:**

To explore whether the diagnostic homogeneity in a daily, routine clinical activity changed visibly over two historical periods (the ICD-8 and the ICD-10 era) across and within five psychiatric in-patient clinics.

**Methods:**

In this register study, we analyzed the discharge diagnoses from five university-affiliated departments of psychiatry in Denmark in two time periods: 1980–1985 (ICD-8) and 2001–2010 (ICD-10).

**Results:**

The synchronic inter-departmental diagnostic differences did not decrease in the ICD-10 era compared with ICD-8 era. Nor did the diachronic stability within each department become more homogeneous.

**Conclusion:**

The diagnostic variability reflected by the diagnostic differences between the departments and by the diagnostic homogeneity within each department remained similar in the two historical periods with no evidence of an increased homogeneity of diagnostic habits after the introduction of the ICD-10.

**Limitations:**

There is a myriad of variables that affects the diagnostic variability over time that we were not able to control.

## Introduction

The release of DSM-IV and DSM-5 made it clear that the operational DSM-III promise and goal of an etiology-anchored classification, failed to materialize [1, 2]. A need for etiological research progress and its prerequisite, the diagnostic reliability, was the justifying and motivating factor behind abandoning a prototype-based classification (e.g. ICD-8, DSM-II) in favor of a criteria-based polythetic operational diagnosis in the DSM-III, its subsequent editions, and ICD-10 [3]. Current realization of etiological stagnation stimulated a lot of criticisms against the contemporary DSM/ICD diagnostic systems, e.g., as lacking validity, being unfit or even counterproductive for research, and with limited clinical utility [1, 2, 4, 5] and an avalanche of theoretical reflections on the nature of psychiatric classification [6–10].

Despite these criticisms, the operational systems, as such, are widely considered as being essentially (epistemologically) sound and as having, indeed, improved the diagnostic reliability in a daily clinical setting. In a series of empirical and conceptual publications [3, 11–14], we questioned the first assumption concerning the epistemological foundations.

In this study, we wish to explore the empirical evidence for the second assumption of an improved diagnostic homogeneity in a daily, routine clinical activity. The available information typically deals with diagnostic reliabilities that are reported as interrater agreements for selected disorders in the so-called “field-trials”, accompanying the construction of diagnostic criteria or in research studies [15–19]. Although both types of reports (field and research trials) stem from somewhat artificially constructed situations, the reliabilities from field studies for ICD-10 and DSM-5 are far from being adequate [16–20]. Most importantly, however, such reports do not provide information on a daily routine reliability across different historical time periods. To the best of our knowledge, there are no published data on the general quality of everyday diagnostic activity (inter-clinician reliability) of different diagnostic systems.

We have, therefore, decided to assess and use the diagnostic variability between and within five similar in-patient facilities in Copenhagen across different time periods as an indirect expression of reliability. To put it simply: if two departments A and B, with similar size, catchment areas, and admission policies, tend to discharge 50 and 20 % of their patients with the diagnosis of schizophrenia, respectively, then one may suspect that the concept of schizophrenia used at these two departments is different.

All five departments are public, free of charge, university-affiliated facilities, each serving residents of a specific geographical catchment area (typically above 100,000 inhabitants). The socioeconomic status and the ethnic composition were similar across the catchment areas, and without major or sudden temporal demographic shifts within the span of each time period.

The variability in the diagnostic assignment may manifest itself as differences between the departments with respect to their distribution of major diagnostic categories at the same point of time or as marked fluctuations over time in the diagnostic distributions within the same department. Although small variability is expectable and multidetermined, a marked variability is typically due to instability and inconsistency in the processes of diagnostic assignment.

We hypothesized that the transition from ICD-8 (a prototypical system, used in Denmark until 1994) to the ICD-10 (a polythetic operational system) would visibly increase the uniformity of psychiatric diagnoses across the five examined psychiatric in-patient departments, with limited inter-departmental differences and result in small yearly fluctuations in diagnostic distribution within a given department. We assumed that the introduction of the ICD-10 diagnosis, based on a specific number of explicit criteria and explicit diagnostic rules, would diminish the space for inconsistency, local idiosyncrasies and subjective preferences, thus improving in the diagnostic rigor and increasing diagnostic uniformity. In Denmark, research criteria of the ICD-10 are used for clinical purposes.

We decided to study the following variables: (1) the distribution of discharge diagnoses in the investigated departments during ICD-8 and ICD-10 eras, respectively, and (2) the temporal stability of the discharge diagnostic distributions within each department.

## Methods

### Setting and sampling

Five psychiatric departments in greater Copenhagen, jointly serving a total population of 812,300 citizens, were selected for the study. Each department serves its own specific geographic area. All departments are general psychiatric, university-affiliated in-patient facilities, with identical service obligations. Each department is a general psychiatric department without a formalized particular profile. The patients are, thus, admitted independently of their diagnostic presentation. The vast majority of the in-patients are acutely admitted. There are no private psychiatric in-patient facilities in Denmark.

Two periods of time were selected: time 1: 1980–1985 (ICD-8) and time 2: 2001–2010 (ICD-10). Data for in-patients’ discharge diagnoses from the departments were obtained from the Danish Psychiatric Central Register [21]. Each patient was only counted once per year, i.e., if a patient was admitted and discharged three times during 1 year, this patient would only count once with the hierarchically highest, main diagnosis.

The discharge diagnoses were stratified into the following groups: (1) schizophrenia (ICD-8: 295, 297.19 and 297.99 and ICD-10: F20–F20.9), (2) bipolar, depression and recurrent depression (ICD-8: 296, 298.09 and 298.19 and ICD-10: F30–F33.9), (3) schizotypal disorder (ICD-8: 301.83 and ICD-10: F21), (4) personality disorders (ICD-8: 300, 301.00–301.99 except 301.83 and ICD-10: F60–F61.9), and (5) other mental illness (including all other psychiatric diagnoses, e.g., primary alcohol or substance abuse, organic disorders, anxiety disorders, adjustment disorders).

### Statistical analysis

All analyses were conducted at group level. We compared the proportion of discharge diagnoses from the different departments. The differences between the two periods were statistically tested by *t* test for equal means, and the levels of significance were Bonferroni-corrected (*p* < 0.01), and to correct this test for differences in the variances, we used the pooled *t* test [when equal variances were assumed (*p* ≥ 0.05)] and the Cochran *t* test [when the variances could not be assumed to be equal (*p* < 0.05)]. We used the proportion difference test, odds ratios and the corresponding 95 % confidence intervals to illustrate the odds for receiving a particular diagnosis in a particular department versus receiving the same diagnosis in one of the four other departments in each time period.

The year-to-year stability within each department was reflected in the homogeneity of the variance (the squares of standard deviation) and was tested by folded *F* test. Finally, we wanted to compare the variances between the two time periods adjusted for department. Therefore, we performed a two-way ANOVA with time period and department as independent factors and all standard deviations from time 1 and 2 as the dependent outcome variables.

## Results

The total number of patients for the five departments was 50,928 over the 6 years of time 1 and 51,899 over 10 years in time 2. The number of discharged patients was nearly the same, whereas the time 1 period was markedly shorter. Moreover, over this historical period, the number of psychiatric hospital beds for time 2, was more than halved. In other words, the “productivity” indexed here by the yearly number of discharged patients, increased dramatically in the ICD-10 era.

Table 1 shows the percentual diagnostic distribution (mean and standard deviation) and the *p* value for the equal variances for the two time periods in each department. The means were significantly different between the ICD-8 and ICD-10 periods (*p* < 0.001) for almost all diagnostic groups and departments. In the ICD-10 era, there was an increase in the proportions of the diagnoses of schizophrenia, affective illness (1.6 times) and of schizotypal disorders (1.5 times). There was a corresponding drop in the diagnoses of personality disorders and other mental illness (2.5 and 1.3 times, respectively, more frequent during the ICD-8 era).

**Table 1:** Percentual diagnostic distribution and test for equal variances in time period 1 and time period 2 in each department

Schizophrenia	Time period 1		Time period 2		Each department, folded *F* ^b^
	Mean_1_	*s*_1_^a^	Mean_2_	*s*_2_^a^	*p* value, testing equal variances
	*N* = 7374		*N* = 12,079		
% in department 1	11.23	1.42	18.80	2.02	0.46
% in department 2	17.27	2.77	28.20	3.27	0.75
% in department 3	10.23	1.18	25.51	2.02	0.25
% in department 4	12.64	1.43	23.88	3.61	0.06
% in department 5	18.51	0.90	20.66	1.99	0.09
% in all departments	13.97	3.70	23.41	4.25	0.43

Schizotypal disorder	Time period 1		Time period 2		Each department, folded *F* ^b^
	Mean_1_	*s*_1_^a^	Mean_2_	*s*_2_^a^	*p* value, testing equal variances
	*N* = 982		*N* = 1495		
% in department 1	2.82	0.98	1.42	0.47	0.05
% in department 2	2.11	0.41	7.63	1.59	**0.008**
% in department 3	1.60	0.58	2.61	0.72	0.66
% in department 4	1.92	0.54	1.61	0.36	0.26
% in department 5	1.81	0.47	1.68	0.47	1.00
% in all departments	2.05	0.72	2.99	2.51	**<0.0001**

Affective disorders	Time period 1		Time period 2		Each department, folded *F* ^b^
	Mean_1_	*s*_1_^a^	Mean_2_	*s*_2_^a^	*p* value, testing equal variances
	*N* = 7336		*N* = 11,996		
% in department 1	19.95	1.29	26.89	1.90	0.41
% in department 2	14.09	1.33	21.40	3.75	**0.03**
% in department 3	12.17	1.62	19.53	1.27	0.50
% in department 4	18.61	2.43	35.00	2.41	0.93
% in department 5	12.83	0.61	20.54	1.83	**0.03**
% in all departments	15.53	3.52	24.67	6.25	**0.002**

Personality disorders	Time period 1		Time period 2		Each department, folded *F* ^b^
	Mean_1_	*s*_1_^a^	Mean_2_	*s*_2_^a^	*p* value, testing equal variances
	*N* = 5478		*N* = 2249		
% in department 1	11.22	2.49	3.84	0.67	**0.001**
% in department 2	7.21	1.77	3.21	0.86	0.06
% in department 3	9.59	1.21	4.30	0.58	0.06
% in department 4	10.81	1.14	2.90	0.58	0.07
% in department 5	12.74	0.73	6.09	2.14	**0.03**
% in all departments	10.31	2.39	4.07	1.57	**0.009**

Other mental illness	Time period 1		Time period 2		Each department, folded *F* ^b^
	Mean_1_	*s*_1_^a^	Mean_2_	*s*_2_^a^	*p* value, testing equal variances
	*N* = 29,758		*N* = 24,080		
% in department 1	54.77	1.78	49.05	2.30	0.59
% in department 2	59.33	1.84	39.56	1.88	1.00
% in department 3	66.41	3.57	48.05	2.56	0.36
% in department 4	56.02	3.15	36.61	3.49	0.87
% in department 5	54.11	1.22	51.02	2.87	0.07
% in all departments	58.13	5.13	44.86	6.29	0.24

^a^ *s*_1_ and *s*_2_ are the standard deviations of the average percentage (mean_1_ and mean_2_) in time periods 1 and 2 for a specific diagnosis and a specific department

^b^The variances *s*_1_^2^ and *s*_2_^2^ are compared using the folded *F* statistic, *F*′ = max (*s*_1_^2^,* s*_2_^2^)/min(*s*_1_^2^,* s*_2_^2^) for each diagnosis and department combination

**Fig. 1:**
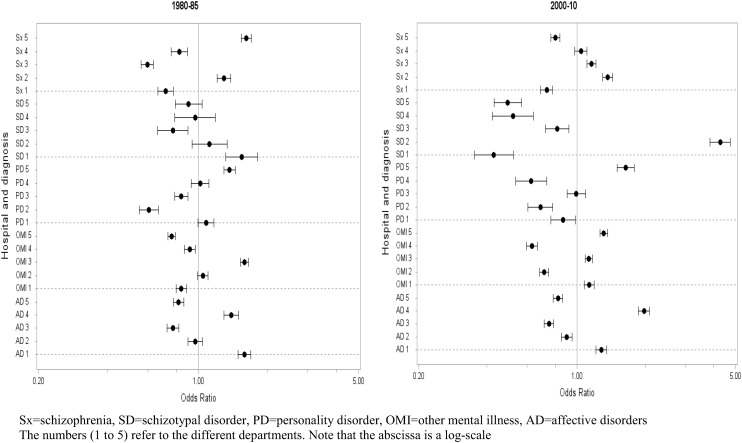
Odds ratio with 95 %-confidence intervals for receiving a particular diagnosis in a particular department versus receiving the same diagnosis in one of the four other departments

Figure 1 shows, separately, for each time period, the odds ratio and 95 %-confidence interval for the likelihood of receiving a particular diagnosis in a particular department versus receiving this same diagnosis at any of the other four remaining departments. There was no general tendency for smaller odds ratios in time 2 (ICD-10) compared with time 1 (ICD-8). In other words, there was no reduction of inter-departmental diagnostic variability in the ICD-10 era.

Table 1, last column displays the *p* value from “testing equal variances”. *p* values in bold are significant (*p* < 0.05) and indicate a change in the variance from time 1 to time 2, i.e., a significant change in the year-to-year stability. The stability did not change significantly for schizophrenia in any of the five departments, while it decreased for affective disorders in two of the five departments. For personality disorders, the year-to-year stability also became more unsteady in the ICD-10 period in one department, more stable in another and unchanged in the remaining three departments. For schizotypal disorder, one department showed more year-to-year fluctuation during the ICD-10 period. For ‘other mental illness,’ there were no significant changes in the year-to-year stability between the ICD-8 and ICD-10 periods. No significant change was detected for any of the five diagnostic groups in a two-way ANOVA with time period and department as independent factors and all standard deviations from the time 1 and 2 as the dependent outcome variables. We could not assess the interaction term between department and time period because of insufficient number of the degrees of freedom.

## Discussion

In viewing the results of the study, the reader must bear in mind that the profiles of discharge diagnoses are dependent on a myriad of socioeconomic, medical, bureaucratic, and other factors which are not controlled in the present report. Consequently, the proportions and the variations are only indirectly related to the potential effect of a given diagnostic system.

The study spans over a historical period, which had witnessed major reductions in the number of psychiatric beds and a concomitant expansion of community mental health services. These changes affected equally the studied departments and their catchment areas, thus having limited influence on the results for period 2. Given an increase in out-patient facilities, one could, perhaps, assume that psychotic patients were less likely to be hospitalized during period 2. However, the yearly number of discharged patients increased dramatically in the ICD-10 era. This is consistent with our clinical experience that the vast majority of psychotic patients become admitted at a certain point at the hospital.

The proportion of non-Danish residents was higher during the ICD-10 era, possibly influencing the diagnostic distribution for that period of time, but without direct bearing on our research questions. We had no possibility to examine a potential influence of the density of non-Danish patients in a given catchment area for the diagnostic distributions. The incidence of schizophrenia in Denmark was declining or stagnant until the late 1980’s [22]. Thereafter, it began to rise [23]. In addition, multiple pilot feasibility studies preceding systematic research projects conducted in Copenhagen from the mid 1990’s to current, suggest a rather monotonous or slightly increasing incident schizophrenia spectrum disorders [24–26].

Our main hypothesis was that the introduction of ICD-10 research criteria for clinical use (as it was the case in Denmark) would increase the diagnostic uniformity between the different departments. In other words, we expected more limited diagnostic differences between the departments in the ICD-10 era. The picture which emerged was, however, much more ambiguous. In some departments and for some diagnoses, the differences became smaller, whereas for other departments and diagnoses, the reverse was true. No general tendency of ORs approaching 1 (reflecting increased uniformity) from time 1 to 2 was observed for any of the diagnoses (Fig. 1).

An interesting finding is the large odds ratios for schizotypal disorder in the ICD-8 era for departments 1 and 2, continuing in the ICD-10 era only in department 2. Behind these numbers is a story involving specific research interests and traditions. The concept of “schizotypy” for clinical use was introduced in Denmark in 1970s by a psychiatrist and psychoanalyst, trained in the US, who was a leading physician at department 1 and strongly influenced its diagnostic practice [27, 28]. He also trained the psychiatrists who eventually came to occupy senior positions at department 2 during the examined ICD-8 and ICD-10 periods. This latter group engaged in a continuing schizophrenia-oriented research at department 2 in the ICD-8 and ICD-10 periods, with an emphasis on the concepts of the schizophrenia-spectrum and schizotypy, e.g. US-DK adoption studies, Copenhagen linkage and high-risk studies, and most recently, studies on the self-disorders in schizophrenia spectrum disorders [29–36]. The research interests of department 1 changed in the ICD-10 period to new directions, unrelated to schizophrenia. We think that the phenomenon of the relationship between a department’s research profile and its diagnostic habits deserves further study in a more systematic way.

We expected that the introduction of ICD-10 would increase the year-to-year diachronic stability of the diagnostic distributions within each department. The variability either increased or remained unchanged for all five diagnoses in all departments except for personality disorders, where the variability decreased in one department from time 1 to time 2. These results suggest that the ICD-10 period was not associated with a decrease in variability of diagnoses.

Our finding of significant differences in the proportions of the diagnostic groups between time 1 and time 2 was, of course, not surprising, because different sets of diagnostic criteria were applied (ICD-8 and ICD-10). For example, the diagnosis of schizophrenia in the ICD-10 requires one month’s duration, whereas it was a Danish ICD-8 clinical rule to use the schizophrenia diagnosis mainly for a chronic disorder with severe negative symptoms [32]. Similarly, the increase in affective disorders may be ascribed to a marked sensitivity of the ICD-10 concept of “major depression” [37].

The proportion of personality disorders and other mental illness became smaller during the ICD-10 period. This is likely due to a more explicit availability of a syndromic diagnosis (aka Axis I) in ICD-10 but may also be related to increasingly scarce number of psychiatric beds. This may have elevated the hospitalization threshold, thereby shifting the diagnostic distribution toward more serious conditions.

Several factors limit the study: it is well known that psychiatric departments often have different “diagnostic cultures”, irrespective of the official diagnostic system in use. Such differences are due to a great number of factors, e.g., varying degrees of interest and competence in the study of psychopathology, particular research programs involving specific diagnostic groups, psychotherapeutic interest and tradition and even quite mundane issues such as the turn-over speed or shortage of psychiatrists and other mental health professionals (vacant positions) [12]. It is, of course, a weakness of this study that we were unable to explore the multitude of relevant factors operating behind the presented numbers.

Psychiatric diagnosis is essentially based on clinical description, and it seems to us that a uniformity of diagnostic practice can only be assured by a systematic study, training and teaching of psychopathology [12–14, 38, 39].

## Conclusion

We examined the discharge diagnostic distributions between five psychiatric departments in Copenhagen during the ICD-8 and ICD-10 time periods. We looked at the synchronic inter-departmental differences and diachronic stability within each department. We found no evidence of an increased homogeneity of diagnostic habits after the introduction of the ICD-10.
